# Investigation of Unresolved Interface “Rag Layer” in Athabasca Oil Sand Bitumen *In Situ* Recovery

**DOI:** 10.1021/acsomega.4c04946

**Published:** 2024-07-11

**Authors:** Evgeniya Hristova, Stanislav R. Stoyanov, Richard McFarlane, Kasra Nikooyeh

**Affiliations:** † Natural Resources Canada, CanmetENERGY Devon, 1 Oil Patch Drive, Devon, Alberta T9G 1A8, Canada; ‡ InnoTech Alberta, 250 Karl Clark Road, Edmonton, Alberta T6N 1E4, Canada

## Abstract

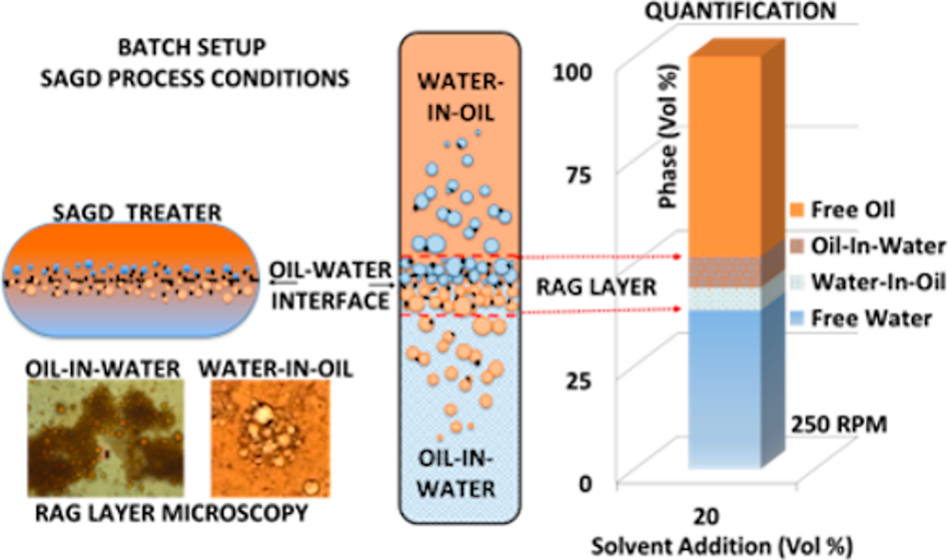

Steam-assisted gravity drainage (SAGD), the leading commercial in situ bitumen recovery process, involves the underground injection of steam and produces at the well head a hot fluid containing water, hydrocarbons, and sand. This fluid is subjected to separation by diluent addition and gravity in several parallel treaters. Occasionally, the separation may be disrupted in one or few treaters by the occurrence of an unresolved interface or “rag layer” while continuing without disruption in the rest of the treaters. In the current study, we investigate “rag layer” occurrence based on the quantification of laboratory-scale and SAGD field tests and imaging of the “rag layer” morphology. The quantification results show that the formation and volume of the “rag layer” are affected by solids, mixing speed, and solvent addition. The microscopic images demonstrate the presence of both water-in-oil or oil-in water emulsions with a distinct transition between the continuous phases. The visual detection boundaries of the “rag layer” are defined as the threshold between the agglomerated and individual droplet layers. The extent of agglomeration increases in the proximity to the oil–water interface. The contribution of hydrophobic fine inorganic solids (less than 10 μm) to forming a “rag layer” is supported by their accumulation observed at the treaters’ oil–water interface, compared to the feed. In well-controlled field operations, the perceived randomness of “rag layer” occurrence could be associated with the fluctuation of fine solid contents in the feed.

## 1 Introduction

**Figure 1:**
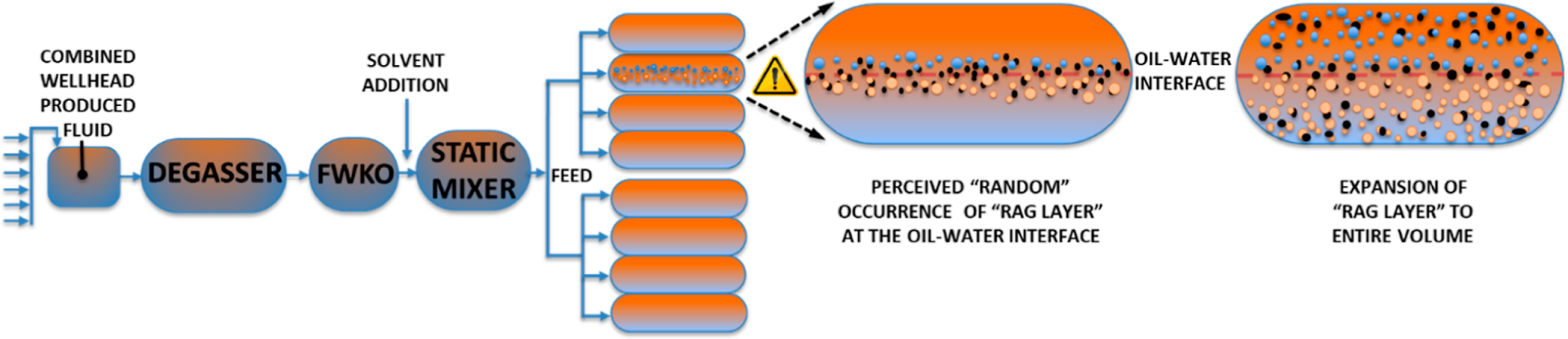
Schematic diagram of a SAGD ground facility with the occurrence of a “rag layer” in one of the treaters. FWKO = free water knockout unit.

Western Canada’s Athabasca region holds the world’s third largest hydrocarbon deposits in the form of oil sand bitumen.^1,2^ A very heavy petroleum, bitumen, is recovered from shallow deposits^3^ by surface mining and from deep deposits using in situ processes, such as steam-assisted gravity drainage (SAGD). Since 2013, the annual production of bitumen recovered in situ has consistently exceeded that from mining.^4^ The SAGD commercial in situ process is based on the use of steam injection through a horizontal well to deliver heat to the reservoir. Bitumen is produced through a second horizontal well, situated below the injection well, and pumped to the surface well pads as a hot produced fluid emulsion (∼200 °C), also containing a water phase (∼80%) and residual sand (∼5–10%).^5−7^ The produced fluid streams from multiple wells are combined and sent to a ground central processing facility (Figure 1), where the main goal is to separate the valuable bitumen product from water and sand and treat the produced water for reuse.^8^ In the degasser and free water knockout (FWKO) stages, the volatile components are captured, and the water content is reduced from ∼80 to ∼30% by volume. Also, in the FWKO stage, most of the coarse sand is removed.^9^

The densities of bitumen and water are very close (∼1 g/cm^3^ at ambient conditions); thus, their further separation is assisted by solvent addition that is intended to dissolve bitumen and increase the density difference between the water and hydrocarbon phases.^10^ Dewatering agents are also added to the produced fluid to facilitate phase separation. Several horizontally elongated vessels, known as SAGD treaters, are connected in parallel to the same feed train and used to allow diluted bitumen–water phase separation by gravity. The SAGD treater retention time is about 1 h with a continuous feed and a temperature of ∼120 °C.^11,12^ The lighter diluted bitumen product is collected at the top-end of the SAGD treater, while the denser water phase and remaining fine solids are collected at the bottom, and subsequently subjected to water treatment and recycling.^13^

Occasionally, in one or several SAGD treaters connected to the same feed train, the oil–water separation can be disrupted by the formation and accumulation of an unresolved interface, referred to as “rag layer”;^14,15^ hence, the occurrence may be perceived as “random” as the oil–water separation process continues without disruption in the rest of the treaters.^16^ In the diluted bitumen recovery literature, the term “rag layer” is often used when discussing the poor separation of heavy crudes and water, and its occurrence is associated with stable emulsion formation and droplet agglomeration.^15,16^ This issue is initially noticeable at the oil–water interface, but if not dealt with in a timely manner, it can potentially occupy the entire vessel volume, halting the SAGD treater operation, Figure 1.

Water-in-crude oil (W/CO) emulsions are usually very stable and difficult to remove.^17^ These emulsions are very well recognized^18,19^ to cause problems in the petroleum industry and have to be dealt with at industrial scale. Residual emulsified water droplets present corrosion issues due to the dissolved salt (e.g., NaCl). Residual fine solids have detrimental impacts on pipelines and downstream upgrading and refining processes through pipe erosion and plugging catalyst beds. For in situ recovery processes, such as SAGD, emulsions are likely formed when the produced fluid flows from the reservoir to the well head, or experience turbulent flow through chokes and valves, and especially in centrifugal pumps. A typical colloid science approach would suggest that surfactants, such as naphthenic or humic acids and their salts present in bitumen, could cause emulsion stabilization.^20,21^ Surfactant-based emulsification is addressed by the addition of dewatering agents. Emulsion stabilization could also be caused by the high asphaltene content (≈16–18 wt %) of Athabasca bitumen, which influences the behavior of the water-diluted bitumen emulsions. In the literature, the widely accepted paradigm claims that water-in-crude oil emulsions are almost exclusively stabilized by asphaltenes.^22−24^ This stabilization is attributed to changes in the rheology of the thin liquid film^25^ in the contact zone between adjacent water droplets from fluid-like Newtonian to gel-like non-Newtonian,^26^ rather than the surfactant-like behavior of asphaltenes.^27,28^ Recently, Hristova et al.^29^ have attributed the notable non-Newtonian film rheology to the transition of the oil-continuous phase to a gel-like formation in the contact zone, essentially due to the surface asphaltene precipitation without any noticeable bulk asphaltene precipitation. Moreover, Hristova et al. claim that surface asphaltene precipitation may be initiated below the critical dilution for bulk asphaltene precipitation.^16,30,31^ This is particularly important for SAGD operations, where care should be taken to avoid asphaltene precipitation.

Masliyah et al. also claim that among the most important oil–water emulsion stabilizing agents are fine solid particles,^7^ a problem which is still standing. The ability of the surfactants and solids to stabilize emulsions is governed by their relative affinities for water and oil phases. The largest difference between surfactants and solids as emulsion stabilizers is the source of their energy of attachment to an interface. While for surfactants this energy originates from the lowering of interfacial tension, solids do not lower the surface tension but the free energy of the system, with the largest contribution arising from the immersion in the water–oil interface, essentially replacing it with the particle cross section.^7,32^ The fine solids capable of stabilizing emulsions are mainly inorganic clays, which are naturally hydrophilic but possibly become modified to partially hydrophobic. Hristova et al.^30^ also highlighted the effect of the presence of water-wet solids that improve oil–water separation, while the presence of oil-wet solids impedes the separation performance. Moreover, they evaluated the effect of the controllable parameters on the oil–water separation performance, focusing on the process conditions of a “rag layer”-free interface, and proposed a separation efficiency index that facilitates the oil–water separation efficiency evaluation. The results show that the addition of a solvent to bitumen has a more dominant effect on separation than the mixing rate. This observation highlights the tremendous complexity of the oil–water emulsion stabilization mechanisms.

The “rag layer” occurrence has been extensively studied at conditions relevant to bitumen froth treatment, where it has been generally associated with bulk asphaltene precipitation, solvent addition, and the presence of solids.^14,15,33,34^ Investigations of “rag layer” occurrence and composition under the conditions of in situ recovery are scarce, highlighting the need to understand and address this disruptive process under the conditions of the predominant oil sand recovery process.

In the current study, we investigate the “rag layer” formation that can become disruptive when it extends beyond the oil–water interface and occupies a large part of the treater volume. This study builds upon the recently gained knowledge about oil–water separation efficiency.^16,29,30^ The experiments are conducted at a bench scale, using produced fluids originating from SAGD field facilities in a custom-built setup under process conditions, as well as in a SAGD field sampling activity over several days during “rag layer” occurrence. The unresolved interface visually appears broadened and has a diverse morphology consisting of both oil-in-water and water-in-oil emulsions with a distinct transition between the continuous phases. The amount and composition of the “rag layer” in terms of fine solids and residual oil and water content are correlated with the solvent addition, mixing rate, and solid properties. The findings aim to enable the development of innovative “rag layer” remediation and prevention strategies that could further improve the SAGD oil product quality.

## 2 Materials and Experimental Setup

### 2.1 Materials

#### 2.1.1 Bitumen

The produced fluid samples containing bitumen, water, and solids were obtained from the degasser outlet of a SAGD ground facility. The bitumen was centrifuged for 72 h and 3200 rpm and then filtered through a 5 μm filter at 150 °C to remove any free water and residual solids. The bitumen composition after centrifugation and filtration was 9.7 wt % water and 0.04 wt % solids. The bitumen viscosity was 10,000 cP at 50 °C and density was 1003 and 939.7 kg/m^3^ at 20° and 120 °C, respectively. Before each experiment, the bitumen was rehomogenized for consistency.

#### 2.1.2 Solvent

Commercial natural gas condensate was used as a diluent. Gas condensate is commonly used for solvent addition in SAGD due to its availability in high quantities and economic viability. The gas condensate contained 0.0, 2.0, 37.6, 19.9, and 40.3 wt % of C_3_, C_4_, C_5_, C_6,_ and C_7_+, respectively. Based on the mass fractions, the average carbon number of the gas condensate was 5.98, which was approximated as pseudo-C_6_, and the calculated absolute density was 674.4 kg/m^3^ at 20 °C. The solvent addition range was selected from 10 to 50 vol %, relative to bitumen, in 10% increments to envelop the typical SAGD addition rates of about 30%.^8,30^ The dilution range was below the critical dilution of asphaltene precipitation, determined at 50 vol % for the gas condensate,^16^ as in SAGD operations care is taken to avoid asphaltene precipitation.

#### 2.1.3 Solids

The solid content from the SAGD treater feed, observed in a multiday sampling field activity, ranged from 0.2 to 3 wt %. The intermediate solid content in the unresolved emulsion/suspension of the oil–water interface was 0.42 wt %. This value was selected for the subsequent bench experiments.

The solids used for addition in the bench-scale experiments were fine ground silica (99.2% SiO_2_) with particle size of <5 μm. Two different types of solids in terms of wettability were used for the experiments: untreated quartz powder water-wet by origin,^35,36^ from the feed and quartz powder treated to render it oil-wet. The quartz powder treated to render oil-wet was prepared by soaking for 2 h in a 5% solution of dichlorodimethylsilane in toluene, followed by filtration, sequential rinsing with toluene, acetone, toluene again, and deionized water, and drying. The wettability of these solids has been reported and discussed by Hristova et al.^30^

The field solids were sampled from the SAGD treater inlet for the feed and the treater outlet at the oil–water interface for the “rag layer” and analyzed in the InnoTech Alberta laboratories. The mineralogy was determined using X-ray-diffraction, and particle size distribution (PSD) was determined using laser diffraction and reported by particle number and volume.

#### 2.1.4 Dewatering Agents

All experiments were conducted in the presence of a predetermined constant amount of proprietary SAGD additives, consistent with SAGD field operations. The use of these dewatering agents was necessary to achieve a separation performance representative of that achieved in the field, as determined during commissioning.^30^

### 2.2 Experimental Setup

**Figure 2:**
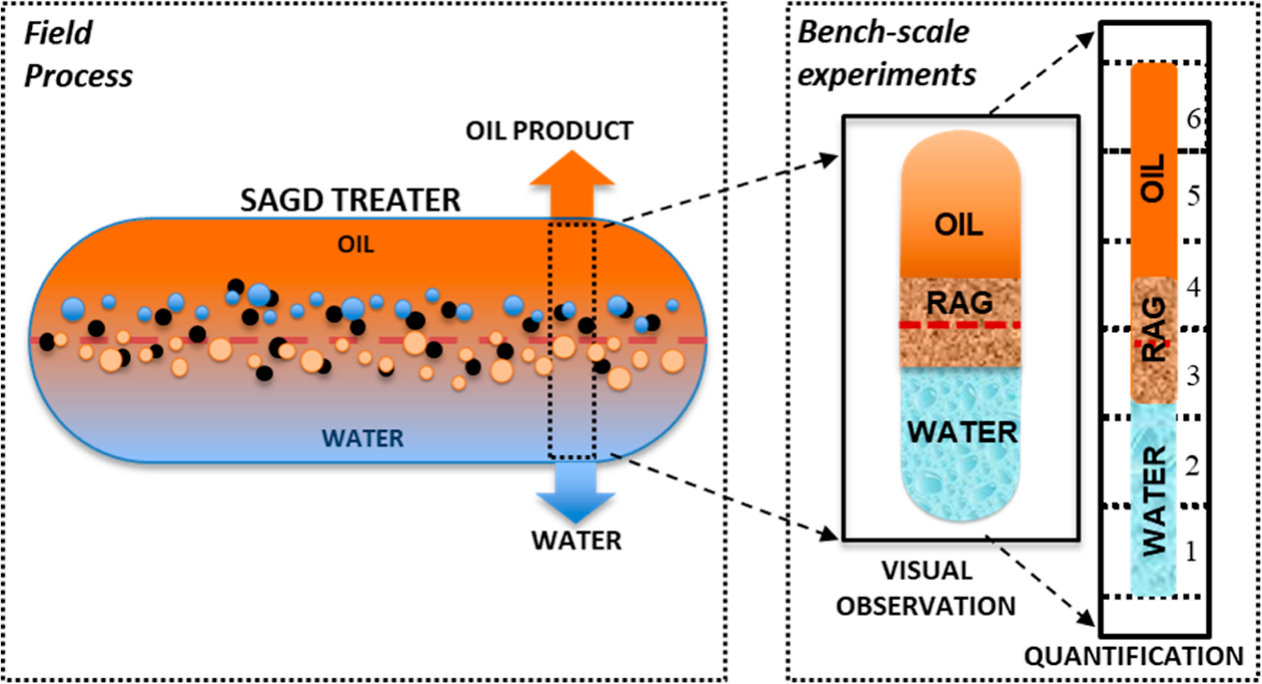
Schematic of a field process SAGD treater and bench-scale experimental setup. Oil–water interface is shown as a red dashed line. In the SAGD treater, the presence of water droplets (blue), oil droplets (in ochre-brown), and solids (black) denotes the “rag layer”.

The factors affecting oil–water separation and occurrence of a “rag layer” in the vertical cross section of an SAGD treater (Figure 2-left) are investigated in a sampling SAGD field activity and in a bench-scale system (Figure 2-right) under process conditions using produced fluids originating from SAGD field facilities. For quantification, the total (Jerguson) cell volume is sampled in six discrete sections. This approach enables the correlation of the results obtained in the field with those from the investigation performed in a controlled bench-scale environment. A particular advantage of this approach is the ability to visually observe the separation and subsequently subsample and quantify the discrete vertical fluid distribution. This allows to evaluate the process at conditions analogous to those of oil–water separation in the field.

### 2.3 Bench-Scale Batch System

**Figure 3:**
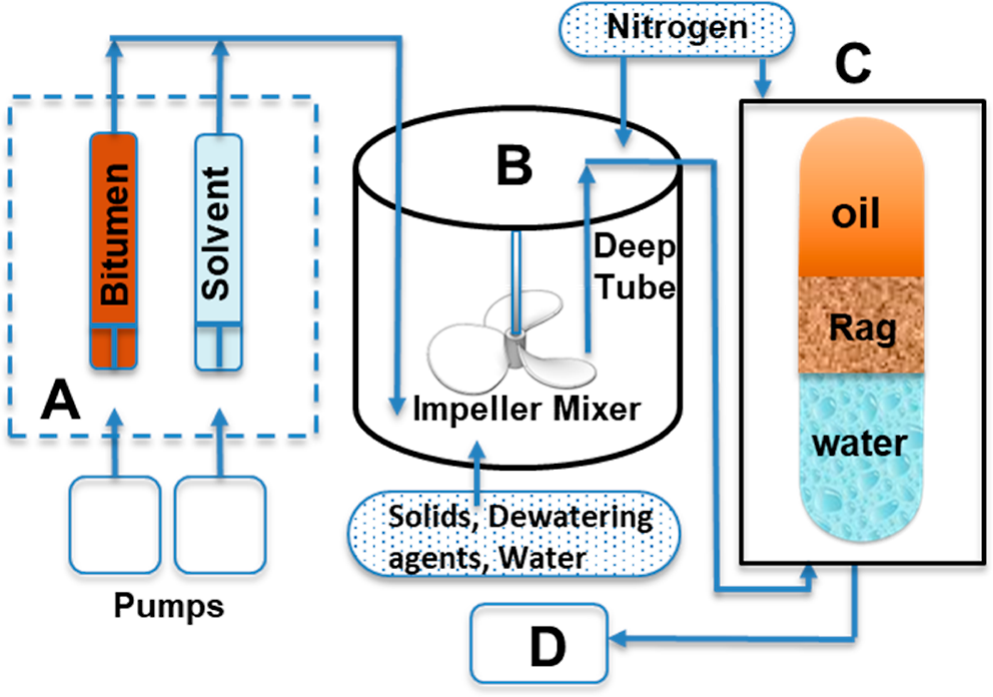
Schematic diagram of batch system apparatus, showing injection (A), mixing and settling in an autoclave (B), visual observation in a pressurized window cell (C), and quantification (D).

The bench-scale batch system (Figure 3) was custom-built to represent the SAGD treater under process conditions. The apparatus is composed of four systems: (A) fluid and addition, (B) mixing, (C) separation and visual observation, and (D) phase quantification. An ISCO syringe pump by Teledyne ISCO, Inc. and stainless steel accumulators (A) were used to meter the solvent and SAGD fluid into the mixing cell (B). A Parr Hastelloy 1 L autoclave (B) with a three-blade propeller-type mixer (diameter 10 cm; impeller size 7.5 cm) was used to provide a variable mixing shear rate (controlled by the variation of the mixer rpm) and a pressurized, heated environment for solvent mixing and the settling of produced fluid blends with the solvent. A Jerguson high-pressure window cell was used to accommodate the visual observation of the gravity separation of the water and oil phases in each experiment. The windows of the Jerguson cell were treated with a hydrophilic agent prior to each experiment to prevent the coating of its sapphire surface with a dark oil phase. To allow for the subsampling of the experimental material, the bottom of the Jerguson cell was fitted with sampling ports designed to avoid additional emulsification during sampling. The obtained discrete vertical layer subsamples then undergo follow-up quantification procedures for the residual water or oil, respectively. The batch system was heat-traced and insulated, and the components were connected through heated lines in order to maintain the required elevated temperature. The top of the Jerguson cell and the autoclave were connected to a pressurized nitrogen supply line to maintain the pressure in the experimental setup and facilitate fluid displacement and drying. The experiments were conducted at the temperature of 115 °C and the pressure of 1100 kPa, conditions representative of those in a SAGD treater.^13^

A series of experiments were conducted to commission the newly assembled apparatus, with special attention dedicated to the mixing system during commissioning.^30^ In a glass vessel with the same geometry as the autoclave, visualization experiments were performed at room temperature and pressure to examine and select a suitable range of mixing conditions. A substitute fluid (silicon oil) with a specific viscosity similar to the bitumen viscosity under process conditions and a water-soluble dye were used to facilitate the visual contrast between the phases.

#### 2.3.1 Experimental Flow

The autoclave was charged with bitumen, water, solids, and dewatering agents at the selected composition and held to the process conditions. Then, a diluent was added at 6.67 cm^3^/min and mixed at the target shear rate. The mixing was initiated in the autoclave at process conditions with diluent addition, and after the required mixing time of 15 min, the impeller was stopped to allow the mixture layers to separate under gravity for 1 h. The layers were then transferred through the dip tube at the bottom of the autoclave to the visual observation system. Mixing rates of 250, 300, and 400 rpm were selected based on commissioning tests, as described by Hristova et al.^30^ A transferring procedure was devised to avoid additional emulsification while the cell was transferred to and from the Jerguson cell. In order to avoid turbulent flow and additional emulsification, nitrogen gas was used to equalize the pressure before the fluid transfers. The transfers were controlled by gradually (slowly) adjusting the pressure difference. The flow-through needle or bow valves were carefully monitored to ensure a laminar flow regime. The Aspen HYSYS simulation software was used to determine the composition and density of the vapor at equilibrium with the mixture at 115 °C, based on high-temperature-simulated distillation data for the bitumen samples. The total volume of each required solvent was calculated by the addition of the content of each component in the liquid and gas phases and their densities at the injection temperature and pressure. The mass balance of the autoclave experiments achieved a value of 98% or higher.

#### 2.3.2 Quantification

The vertical fluid distribution was quantified by applying the following evaluation procedure to each layer: the total volume of the fluid in the visual observation cell (360 mL, Figure 2-right) was sampled into six centrifuge tubes (60 mL each), from the first being the bottom layer to the sixth being the top layer. To induce water–oil separation, the sample content of each tube was mixed with an equal volume of toluene, followed by 1 h of centrifugation at 1800 rpm and 222 *g* force. In some cases, the centrifuge g force used was not sufficient to break the entire emulsion, and some residual “rag layer” was visible. Such samples are subjected to a repeated extraction until the phases are fully separated and quantified. Dean–Stark and Karl Fischer analyses were performed after centrifuging to quantify the composition of each layer and obtain the amount of residual water and water, respectively.

In the case of ideal separation, the water and oil phases are free from residual components. The “rag layer” was calculated as the difference between the ideal (100 vol %) separation and the actual separation measured by the residual water content in the oil-continuous phase and oil content in the water-continuous phase. These calculations were performed for each of the six discrete vertical fluid distribution sections (Figure 2, quantification panel) to reconstruct the percent volume of “rag layer”.

## 3 Results and Discussion

### 3.1 “Rag Layer” Occurrence Observation

**Figure 4:**
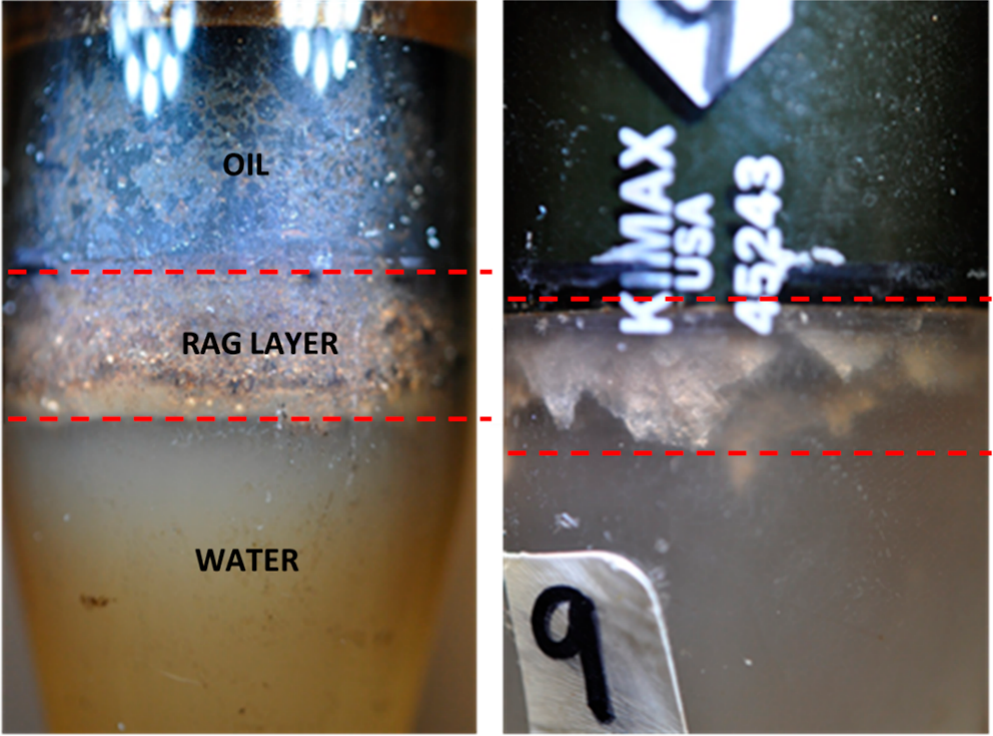
Centrifuge tube containing a “rag layer” (between the red dashed lines) at the oil–water interface of the produced fluid from SAGD treater process conditions subjected to 1 h gravitational separation (left) and after induced separation following toluene addition and centrifugation (right).

For observational purposes, Figure 4 (left) shows an image with an example of a poor separation illustrating the oil–water interface of the produced fluid from SAGD treater process conditions subjected to 1 h gravitational separation. A distinct dense-looking intermediate zone, referred to as “rag layer”, is evident at the oil–water interface between the oil (top) and water (bottom) phases. By visual observation, the appearance of this layer is “muddy”, likely due to elevated amounts of residual oil and solids, as further confirmed by microscopic and composition analyses. It is important to note that the free water at the bottom also appears cloudy due to residual oil or solids, which is additional evidence of poor separation. In Figure 4 (right), the same sample is shown after the induced separation with toluene addition and centrifugation (as described in the Materials and Experimental Setup section). It can be seen that even as the free water layer at the bottom visually appears clear and transparent and the “rag layer” appears thinner, it remains partially unresolved after centrifugation. This indicates the high degree of stability associated with the “rag layer” occurrence, and the above observations are the subject of further evaluation in this study.

### 3.2 Unresolved Phase Imaging/Morphology

**Figure 5:**
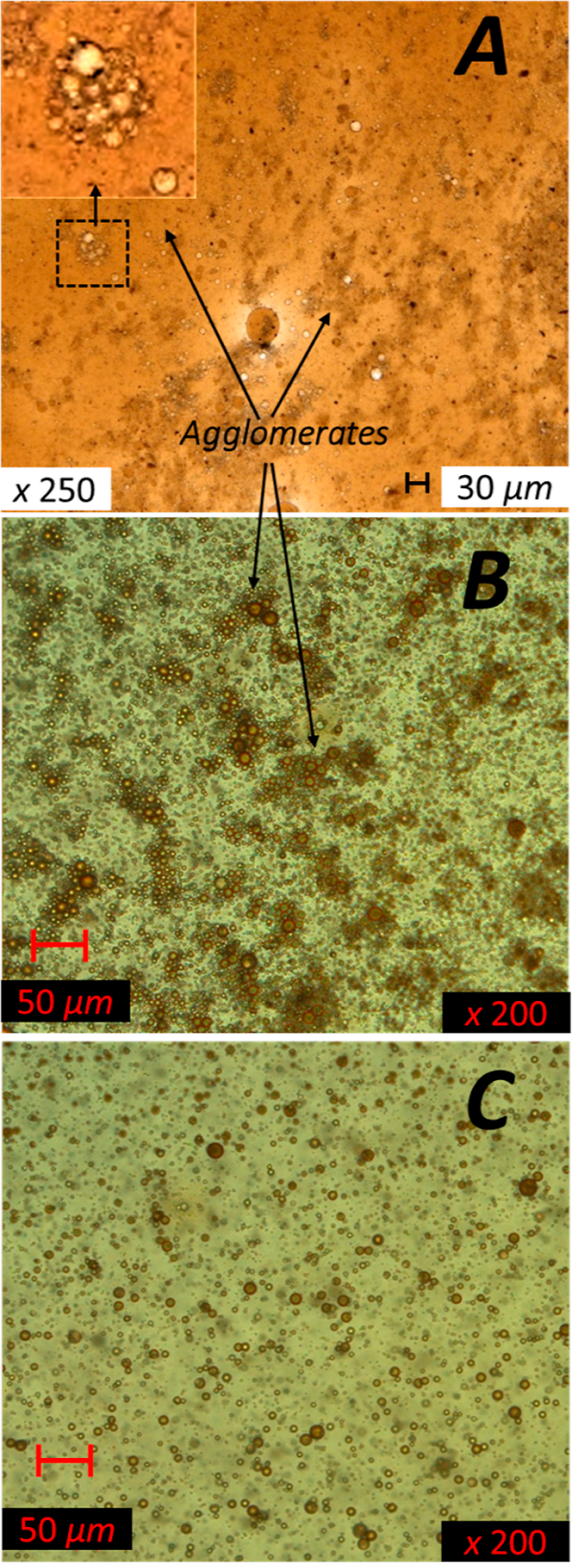
Optical microscopy images of unresolved interface “rag layer” after 1 h of settling, subsampled at different vertical levels: (A) above the interface in the oil-continuous phase with emulsified water droplets (in pale brown color) and suspended solids (in black color); (B) just below the interface in the water-continuous phase in the “rag layer”; (C) below the visual “rag layer.”

**Figure 6:**
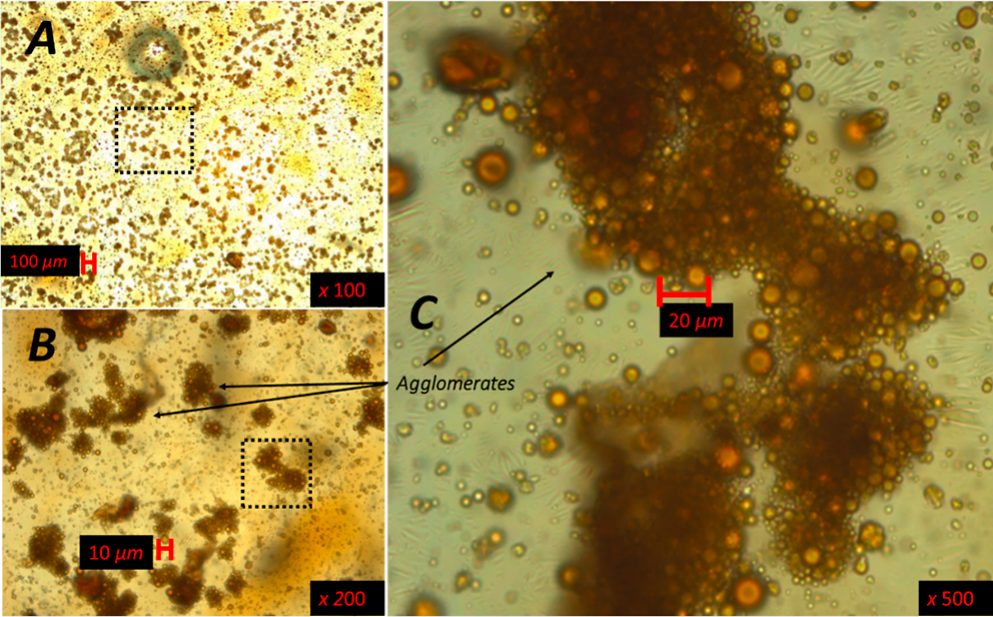
Optical microscopy images of the “rag layer” at different magnifications sampled just below the interface in the water-continuous phase. (B,C) Reimaging approximate areas outlined in dotted rectangles in (A,B), respectively.

The optical microscopy images shown in Figures 5 and 6 provide further insights into the diverse morphologies of these unresolved phases of the “rag layer”. Figure 5 shows a case of poor separation of the produced fluid subsampled from three vertical subsampling levels after 1 h of settling. Figure 5A shows a subsample of a “rag layer”, taken slightly above the oil–water interface, that illustrates the oil-continuous phase (in ochre-brown color) with emulsified water droplets (in pale brown color) and suspended solids (in black color). In the dashed square of Figure 5A, an example of about a dozen water droplets forming an agglomerate is zoomed-in, using the software. A large number of fine solid particles are also seen as both dispersed and attached to the water droplet surfaces within the agglomerate. Upon close observation, all droplets from this image appear to be water droplets, even as some may visually appear ochre-brown as they are covered with a layer of the continuous phase. The partitioning of the fine solids visible only in Figure 5A (i.e., oil-continuous phase) may suggest that they are predominantly oil-wet in this case. The presence of oil-wet solids has previously been associated with impeded oil–water separation.^30^ The subsample in Figure 5B, taken from the “rag layer” slightly below the oil–water interface, features a large amount of emulsified oil droplets (in brown color) in the water-continuous phase, which appear attached to each other, forming agglomerates. Figure 5C is taken further below the visual “rag layer” in the water-continuous phase below the interface. Similar to Figure 5B, emulsified oil droplets are visible in Figure 5C; however, these are mostly individuals, apart from each other, without visible agglomeration. Thus, this fine emulsion does not visually appear within the “rag layer” in the bulk. The differences in the morphology observed in Figure 5 could be related to the distance of the collected sample from the visible oil–water interface. These observations provide insights into the emulsion distribution and show that the extent of agglomeration increases with the proximity to the interface. Thus, the “rag layer” demonstrates emulsion gradient at vertical levels (with variable emulsion type, amount, and degree of agglomeration), with a sharp change in the continuous phase from oil-in-water to water-in-oil emulsion at the interface.

Images of the “rag layer” agglomeration phenomena are presented in Figure 6, where the unresolved interface containing an oil-in-water emulsion is shown at three different magnifications. Analogous to the conditions of Figures 5B, 6A features a substantial number of oil droplets incorporated in agglomerates as well as some single oil droplets. The area inside the dashed line rectangles is acquired at higher magnifications (Figure 6B,C) to show that the agglomerates comprise hundreds of oil droplets with an approximate size of 5 μm or less. The formation of the agglomerates occurs spontaneously, and within the agglomerates the droplets retained their original size with time, i.e., no coalescence is observed. The oil droplet agglomeration is indicative of substantial attractive surface forces acting among them that may play an important role in the stability and behavior of the “rag layer”.

### 3.3 Vertical Distribution Profiles

**Figure 7:**
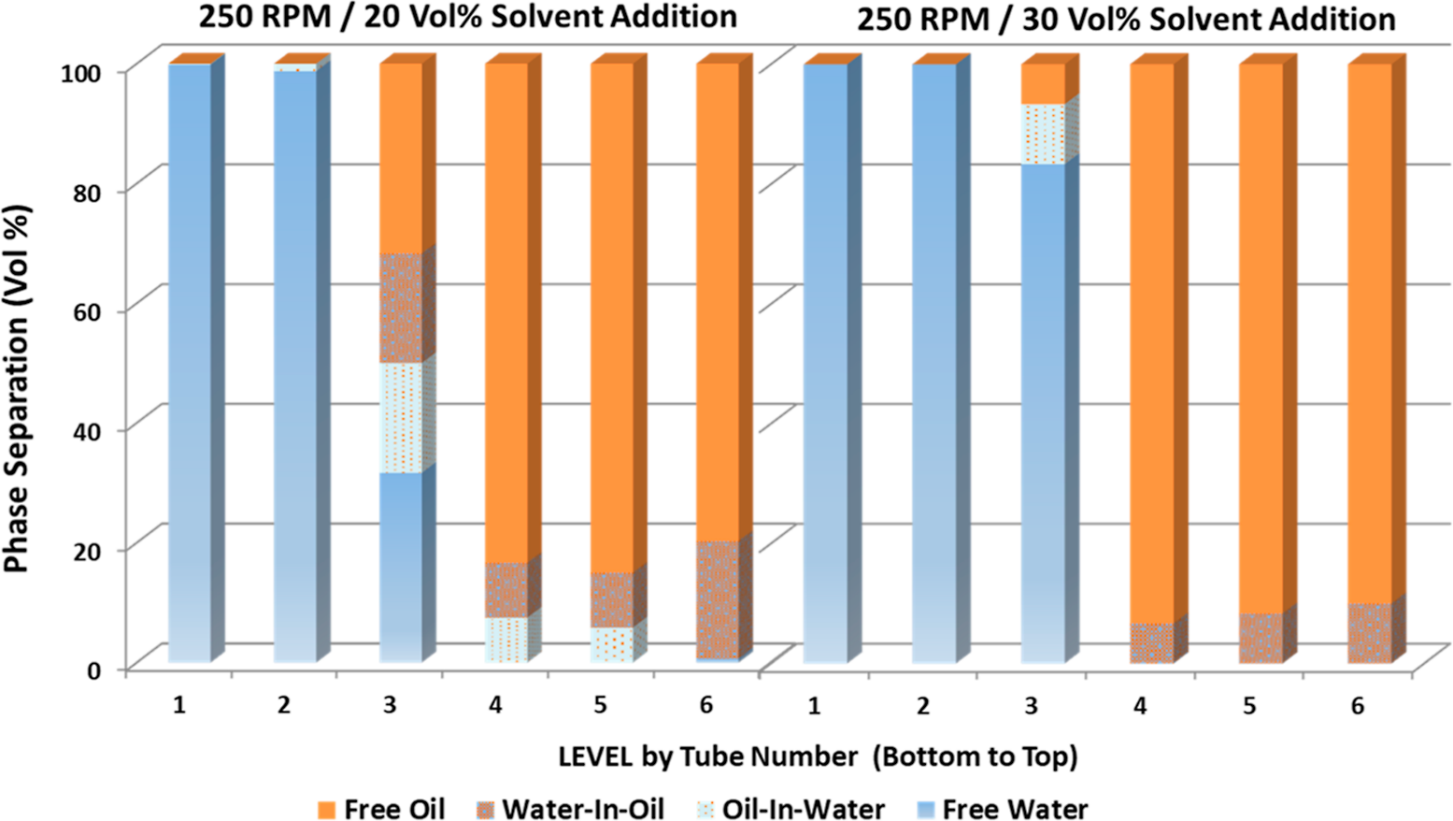
Single-run phase distribution in six discrete vertical levels (1–bottom to 6–top) from the quantification system (Figure 2), representative of poor separation (left, 250 rpm/20 vol % solvent) and good separation (right, 300 rpm/50 vol % solvent).

The oil–water separation efficiency is quantified by determining the volume (mL) of the water and oil phases and assessing the residual water and oil emulsions within each phase. Based on the microscopy observations, the unresolved interface consists of oil-in-water and water-in-oil emulsions (Figures 5 and 6). The experimental observation cell is divided into six discrete levels [bottom to top, Figure 2 (quantification)], and each level is investigated separately to evaluate the phase volumes under a range of mixing conditions and solvent addition. The two examples shown in Figure 7 represent poor (left) and good (right) separation, respectively. In the example of poor separation, the unresolved emulsion “rag layer” is present in all the vertical levels with more substantial amounts close to the oil–water interface (tube number 3) and in the oil phase (tubes numbered 4–6). In the example of a good separation, a small amount of “rag layer” is found at the oil–water interface, while the majority of the produced fluid is reported as containing clear water and oil phases.

**Figure 8:**
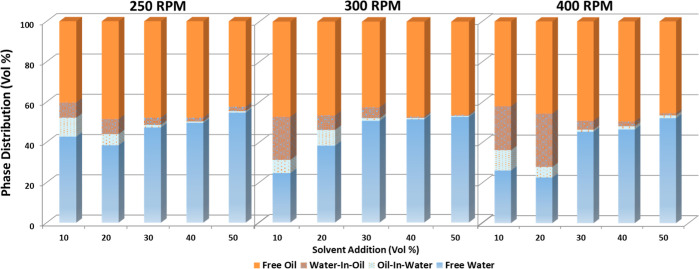
Vertical phase distribution of free oil, water-in-oil, oil-in-water, and free water in the Jerguson cell after 1 h of gravitational settling at varied solvent content and constant mixing rates of 250, 300, and 400 rpm, obtained from the six discrete vertical levels (1––bottom; 6––top) of the quantification system (Figure 2).

In Figure 8, the phase distribution of the entire produced fluid volume is evaluated by quantitatively reconstructing the observational cell and adding the phase distribution volumes of the six discrete vertical levels. The oil-in-water and water-in-oil emulsions and the free oil and water phases are quantified for a range of mixing conditions (250–400 rpm) and solvent addition (10–50 vol %).

The 10 and 20 vol % of solvent addition for all mixing conditions yields a substantial amount of unresolved interface, with the volume of the “rag layer” increasing as the mixing speed increases. This indicates the dominant effect of the mixing speed to generate a stable emulsion at such a low solvent addition. In most cases, the water-in-oil emulsion volumes are substantially higher than the oil-in-water emulsion volumes. It is noticeable that this effect is more pronounced with the increase in the mixing speed. Further investigations are necessary to understand the kinetics of formation and stabilization of these emulsions, such as the evaluation of the effect of the mixing condition on the emulsion morphology and behavior in terms of the emulsion droplet size and amount. For 30 vol % solvent addition, the oil-in-water and water-in-oil emulsion volumes are substantially reduced and become insignificant for 40 and 50% solvent addition, indicating the improved oil–water separation efficiency upon increasing the solvent addition.

These results demonstrate the importance of the solvent addition amount to achieve good separation efficiency and avoid “rag layer” formation, with the threshold of not less than 30 vol % of gas condensate as a diluent. The solvent addition threshold of 30 vol % is attributed to the need to operate SAGD without asphaltene precipitation, as investigated by Hristova et al.^16^ The addition of 30 vol % of gas condensate in SAGD yields optimal separation performance below the critical dilution of asphaltene precipitation, based on the solvent addition rate, shear rate, the presence and wettability of fine solids, and economic viability.^30^ This outcome coincides with the common SAGD diluent addition rates; however, it is important to highlight that any alteration in diluent addition rates, such as localized high solvent concentration or insufficient mixing, may provide suitable conditions and trigger the “rag layer” occurrence, as was also suggested by Hristova et al.^16,29,30^

### 3.4 Effect of Solids

In the field sampling activity, the solids in the “rag layer” and feed were sampled over multiple days and analyzed. The results presented here are intended to highlight the main findings of the field testing relevant to the “rag layer” occurrence with the exception of some specific details due to proprietary restrictions. The solids are characterized as inorganic materials insoluble in the extraction solvent. The mineralogical composition analysis reports more than 70% silica or quartz, with smaller amounts of halite and kaolinite. The solid content in the “rag layer” is 2 to 3 orders higher than that in the feed. These results indicate a tendency of solid accumulation at the oil–water interface in the SAGD treaters. Fluctuation in the feed solid content is also detected, as significantly higher than the average amount of solids reached the treaters on some sampling days. This perturbation could be associated with the perceived randomness of the “rag layer” formation.

In the field sampling activity, the PSD data by laser diffraction reported by particle number have median values (D50) of around 0.60 μm or less. The PSD data reported by particle volume ranged from 2 to more than 100 μm. Comparison between the PSD by number and by volume is an indication of a relatively small number of large particles present in the “rag layer”. This indicates the presence of a large proportion of fine particles (less than 10 μm), which would have very low settling velocities.^37^ Such fine particles could be adsorbed on the diluted bitumen–water interface and contribute to the formation of a “rag layer”.

### 3.5 “Rag Layer” Definition in SAGD

**Figure 9:**
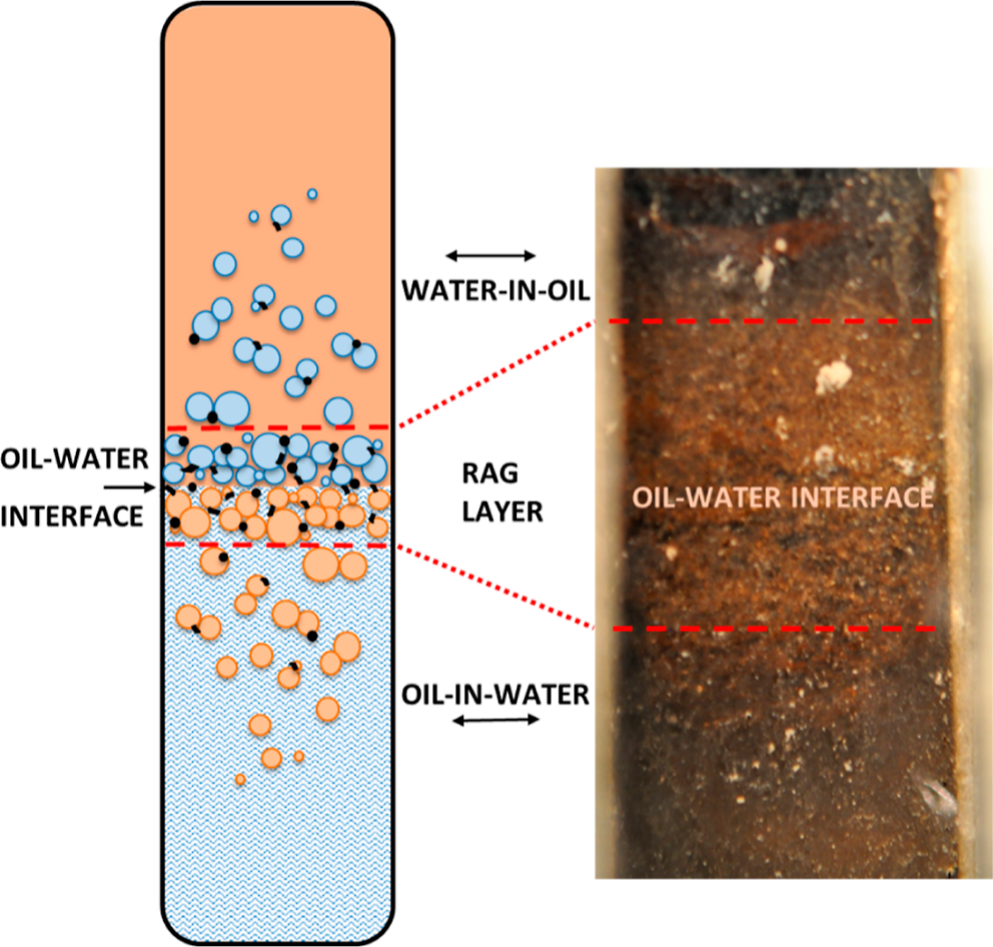
Schematic diagram of a gravity-separated system containing water, solids, and diluted bitumen with a “rag layer” at the oil–water interface aligned with a visual observation window image showing the vicinity of a “rag layer”.

Supported by the findings gathered in this and previous studies,^16,30^ a schematic diagram of a “rag layer” in terms of diluted bitumen recovery is presented in Figure 9 (left) and correlated with an unresolved emulsion image from the observation cell window Figure 9 (right). This diagram defines and highlights the key components of the “rag layer” and contributes to elucidating its nature and occurrence.

#### 3.5.1 “Rag Layer” Nature

In this definition, the “rag layer” is the unresolved interface containing water, fine solids, and diluted bitumen localized near the oil–water interface. This may visually appear as a broadened interface (Figure 9, right, shown as the area between the dashed red lines); however, on the microscopic level, one can observe dense systems containing water-in-oil or oil-in water emulsions with a distinct transition between the continuous phases. The oil–water interface as shown in Figure 9, left with a black arrow (oil–water interface), is always a sharp transition and is defined by the continuous phase, and even these two emulsions visually appear together to form the “rag layer”.

The limit of visual detection of the “rag layer” can be defined as the threshold between the areas containing predominantly agglomerated (Figure 5A,B) and individual droplets (Figure 5C). The comparison in the visual observation of “rag layer” in Figure 9 (right) with the microscopic imaging in Section 3.2 elucidates that an emulsion consisting of individual droplets further away from the interface at the microscopic level may not visually appear as a “rag layer” in the bulk observations. This is an important insight considering that the timely visual detection of the “rag layer” in the SAGD treater is essential to maintain continuous production.

#### 3.5.2 Rag Layer Occurrence

The perceived randomness of “rag layer” formation in only one (or several) SAGD treater(s) connected in parallel to the same feed (Figure 1) is most likely due to the detected solid content fluctuation in the feed and their accumulation at the oil–water interface, which would occur gradually over time. Occasional batches of the produced fluid with higher-than-usual fine content may attribute to the variation in the feed content and uneven distribution to the treaters. For any treater, the probability of “rag layer” formation would substantially increase when a significant amount of solids has been accumulated at the interface.

Another key factor that has been demonstrated to contribute to the “rag layer” occurrence is the hydrophobic affinity of the problematic fine solids. This outcome is in a good agreement with the literature,^7,32^ stating that hydrophobic particles are mainly those capable of stabilizing emulsions, such as “rag layer”, hence affecting negatively (impeding) the oil–water separation performance.

*From a production point of view*, it is important to acknowledge the variety and complexity of the observed unresolved phases, including emulsions and suspensions at the microscopic level, in terms of size, distribution, and degree of agglomeration. It is necessary to highlight the challenges of attempting to make any correlation between the amount of residual oil and water with the visually observed “rag layer” in bulk.

## 4 Conclusions

The nature and occurrence of the “rag layer” were investigated in this study, supported by quantification data from laboratory scale and SAGD field tests. The “rag layer” is defined as an unresolved interface containing water, diluted bitumen, and fine solids localized near the oil–water interface. The “rag layer” may visually appear as a broadened interface; however, on the microscopic level, one can observe a dense emulsion containing both water-in-oil or oil-in water emulsions with a distinct transition between the continuous phases. The formation and volume of the quantified “rag layer” based on phase distribution are affected by solids, mixing speed, and solvent addition. The variations in the “rag layer” morphology could be related to the distance from the visible oil–water interface. In the cases of good separation, the “rag layer” is localized close to the oil–water interface; in the cases of poor separation, it may extend throughout the entire volume and result in process disruptions.

The extent of agglomeration between the emulsion droplets increases with the proximity to the interface, defining the limits of visual detection of the “rag layer” as the threshold between the areas containing predominantly agglomerated and single droplets. Within these limits, the individual agglomerates comprise hundreds of fine (less than 5 μm) oil droplets. The agglomeration occurs spontaneously, and the droplet size is retained with time. This is indicative of substantial attractive surface forces acting among them that may play an important role in the stability and behavior of the “rag layer”. The presence of emulsified single droplets outside the defined visual detection limits highlights the challenges of attempting to correlate the visually observed “rag layer” volume with the actual amount of residual water and oil in the bulk phases.

The contribution of fine inorganic solids (less than 10 μm) to forming a “rag layer” is supported by their accumulation observed at the oil–water interface compared to the feed. The hydrophobic affinity of the problematic fine inorganic solids agrees with the literature in that hydrophobic particles are mainly those capable of stabilizing emulsions, such as the “rag layer”. The perceived randomness of the rag layer occurrence could be associated with the fluctuation in the feed. Occasional batches of the produced fluid with high inorganic fine solid contents, such as those from newly exploited wells, which can deliver higher-than-usual amounts of fine solids, could significantly increase the probability of a “rag layer” formation.

Improved oil–water separation efficiency is achieved by increasing solvent addition, which substantially reduces the oil-in-water and water-in-oil emulsion volumes and helps avoid “rag layer” formation. For the gas condensate as a diluent, the solvent addition should be at or above the threshold of 30 vol %. This outcome is aligned with the common SAGD diluent addition rates; however, it is important to highlight that any alteration in diluent addition rates, such as localized high solvent concentration or insufficient mixing, may trigger a “rag layer” occurrence.
